# Hospital Meetings

**Published:** 1902-02-22

**Authors:** 


### The Institutional Workshop.

### HOSPITAL MEETINGS.

### ST. MARK'S HOSPITAL FOR FISTULA:

Annual Meeting.

This was nominally a meeting of donors and subscribers to St. Mark's Hospital, but the attendance was so small—some half-dozen only being present—that it could not by a stretch of imagination be called a representative meeting. The agenda consisted chiefly of votes of thanks to the various officers of the hospital, all of which were carried as a matter of course No questions of any importance were asked, and the Annual Report was passed without a dissentient voice. At the close of the meeting, which was over in ten minutes, the secretary said that about £300 had been received towards maintenance in response to a special appeal. In the absence of Mr. R. B. Martin, M.P., the chair was taken by Mr. E. Dent.

The report states that the work of the hospital still increases, and that its usefulness is demonstrated by the number of patients, who come from all parts of England, as well as by the surgical report, which shows that both in in-patient and out-patient departments there has been a considerable increase in the numbers relieved during the year. The subscription list, it is pointed out, needs additional names if the work is to be efficiently carried on. The Samaritan Fund, which is controlled by the ladies who are interested in the hospital, has been instrumental in sending many patients to convalescent homes, and in helping others in monetary and other ways. This fund also needs additional subscribers. The following sums have been received from the special hospital funds:—Hospital Sunday Fund, £125; King Edward's Hospital Fund, £100; Hospital Saturday Fund, £47 12s. Contributions have been received from the following City companies: the Worshipful Company of Goldsmiths, Leathersellers, Skinners, Armourers and Braziers, Dyers, Salters (annual subscription, £5 5s.), Saddlers, Carpenters (annual subscription, £3 3s.) and Curriers. Legacies amounted to £544 5s. 11d., and the ordinary income was £2,711 19s. 3d. There is a debt of over £2,000 to the Bankers. Through the kindness of Mr. Arthur Levita, electric light has been fitted in the wards, the out-patient department, and the operating theatre. Weekly gifts of vegetables have been received from Mrs. R. B. Martin. The Right Hon. the Lord Mayor has consented to be president of the hospital during his mayoralty, and to preside at a festal dinner in the Whitehall Rooms on April 24th, when he will be supported by Mr Sheriff Brooks Marshall and Mr. Alderman and Sheriff Bell—£5,000 is the sum which it is hoped to raise by this means.

The Surgical Report states that 457 in-patients were admitted during the year, and that 28 were under treatment on January 1st, 1901. Of these 361 were discharged as cured, 69 as relieved, 4 as unsuitable; 2 were transferred to the out-patient department, and 2 were incurable. Of out-patients, 1,314 were admitted, and 5,867 attendances were made.

A matinée was given on the 11th inst. in aid of the maintenance fund, at the Imperial Theatre, kindly lent by Mrs. Langtry. A special souvenir was designed and prepared, giving "The Story of St. Mark's," and including photographs of some 40 actors and actresses who kindly helped on the occasion, including Mrs. Langtry, Mr. and Mrs. Beerbohm Tree, Messrs. Martin Harvey, Lewis Waller, and others.

### THE LONDON FEVER HOSPITAL.

Statement by Lord Balfour of Burleigh.

There was a very small attendance of Governors of the London Fever Hospital at the annual meeting on Friday, 14th inst., at the Hotel Victoria. This hospital has a committee of from 15 to 25 Governors, which meets monthly, and of those who were present only two were not members of this committee. It may therefore almost be said that the committee adopted the report drawn up by itself. Two explanations of this paucity of numbers and apparently of interest may be adduced; first, a very large proportion of the Governors are not individuals but companies—the managers of all the London hotels subscribe on behalf of their shareholders, and a large number of business houses do the same—and companies are proverbially lacking in conscience. It may be claimed that when a company has subscribed, and has insured a bed in sickness for any of its employees who may contract an infectious disease, it has done all that is required, and that it can hardly be expected to take a personal interest in the way its funds are expended or its employees cared for. To this there may be more than one answer. The other explanation is that the method employed for informing these companies of the date of the meeting is that of advertisement in the daily press, and not by personal summons.

The point of interest about the meeting was undoubtedly the speech of Lord Balfour of Burleigh, President, who reviewed the work of the past year—one of the heaviest the hospital has known, 903 being the total number of cases treated.

How were they Maintained?

Setting aside the 93 who were in the hospital at the beginning of the year, Lord Balfour proceeded to state on what terms the remaining 810 were maintained. Thirty-two were fairly well to do, and paid £3 3s. a week; next came 495 who paid £3 3s. for treatment and nothing more; a small number—11 in the report, 15 according to Lord Balfour—was made up of nurses and wardmaids who contracted the disease in the performance of their duties; these belonged to the class who paid nothing; and the remaining 268, servants of Government and certain employees, were treated free. There was an immense class of persons who were very much averse to casting themselves on the rates, but who would inevitably fall on the rates if they were not helped. These people could not and would not seek admission to the hospitals of the Metropolitan Asylums Board, and the committee felt that in offering to take them for a small payment they were inducing them to leave their dwellings, where they were a source of danger to others, and in this way an enormous amount of good was being done in the prevention of the spread of disease. Some 60 patients were refused for want of space.

Finance.

On previous occasions, Lord Balfour has made some adverse criticisms on the amount granted by the Prince of Wales's Fund. This year, he had a statement to make. Undoubtedly, he said, the inception of this fund tended to take away some subscriptions from individual hospitals. His remarks two years ago were challenged by Sir Savile Crossley, and some little correspondence and a couple of meetings followed, in which Lord Balfour claimed to have proved his case; at any rate, the excellence of the work of the London Fever Hospital was most cordially acknowledged, and the sum of £600 was granted in 1901, a larger sum than in any previous year. "I want to say," Lord Balfour continued, "that even if I did not prove all I thought I should, I am prepared to say that I was most kindly received. The greatest care was taken to examine our statements, and if the same care is always taken, and the same courtesy always shown, the hospitals of London have a very wise friend at the head of that fund. And although I wish for more money, I feel bound to acknowledge with the utmost candour that our application last year was most kindly considered."

Historical.

This year, the speaker went on, completes the hundredth of this hospital's existence and active work. When it began, as a House of Recovery, there was no such institution in London. Typhus fever was then prevalent, and 15 cases were received at 2 Constitution Row, Gray's Inn Road. Fighting its way through much opposition, the House of Recovery attained a position of much prosperity, removing in 1815 to King's Cross. Thence the Great Northern Railway ousted it, and it was established on its present site in Liverpool Road, Islington, in 1849.

For many years the hospital was almost entirely devoted to the reception of pauper patients from the various unions, which paid a capitation grant for each patient sent, until the passing of the Metropolitan Asylums Act, by which provision was made for such patients by hospitals established and supported by the parochial rates. Under these changed circumstances the committee felt that the hospital should be rendered available for persons above pauperism, but who from any circumstances were unable, when attacked by fever, to obtain proper care and attention at their own homes, or whose presence there was a source of danger to others. It is on these principles that the hospital has since been governed, and that a demand exists for such accommodation is evident from the number of patients the hospital receives each year, and from the number to which it is compelled to refuse admission by pressure on its space. During the 100 years in which the hospital has been at work 82,000 patients have come under treatment. The hospital depends in the main on voluntary subscriptions and donations, and though the support accorded to it is well sustained, greatly through the active exertions of the excellent secretary, Major Christie, it does not suffice to carry out the scheme of gradually rebuilding the present hospital and bringing it up to more modern requirements. The land purchased some years ago for the building of a convalescent home, which would relieve the pressure upon the beds in the hospital, is still unbuilt upon, and the committee hope that the public will at no distant date supply them with funds to justify them in carrying out this and other parts of their scheme for improving and extending the work of the institution. One reason for urging this is that under present arrangements the average stay of patients is 37 days, taken over all the various classes of fever; this would not be the case if there were a place to send them to for recovery after illness.

### CHARING CROSS HOSPITAL: ANNUAL COURT OF GOVERNORS.

At Charing Cross Hospital the excellent practice prevails of sending out circulars to all the governors, and also nserting a notice in the daily press. Eighteen governors were present at the annual court this year (at 2 o'clock on Wednesday, 19th inst.), and of these only two were gentlemen representing the annual subscribers. There was a total absence of the ladies whose names appear in the list of governors, and who are therefore entitled to attend.

The New President.

The meeting began with prayer by the chaplain, after which the customary formalities were gone through, viz., the notice convening the meeting and the minutes of the general court of 1901 were read. His Grace the Duke of Norfolk, K.G., one of the vice-presidents, occupied the chair. Mr. Drummond, treasurer, proposed the election of H.R.H. the Princess Louise, Duchess of Argyll, as President, in place of Lord Wantage, who was elected a year ago, and whose death the Council deeply regret. Lord Wantage had been a Vice-President and generous supporter for nearly 20 years, and was intending to preside at the Festival Dinner in aid of the building fund this spring. In memory of her husband's connection with the hospital, Lady Wantage has given £1,000 to the Dinner Fund. The Duchess of Argyll is the first Lady President the hospital has ever had.

The Building Operations.

The next thing on the agenda was the Annual Report of the Council, draft copies of which had been sent out, and which, with the accounts, was taken as read.

The Chairman ran quickly through it, but made no comments. The main point of interest was the building. Last year the Council regretted necessary delays, this year they are able to announce that the work has begun; that the tender of Messrs. Holloway for £82,730 for the first portion of the work was accepted in October, and that H.R.H. the Duke of Connaught will lay the foundation-stone with full Masonic rites at a date to be announced. This portion of the work consists of the new Nursing Home in Chandos Street (in aid of which a most successful entertainment was recently given at the Savoy Hotel), new surgical wards, and new quarters for the resident staff in King William Street, new out-patients' and casualty departments, and the dispensary on the site of Toole's Theatre. For this purpose the sum of £30,000, towards which some £2,000 has already been subscribed, is required; and a Festival Dinner will be held on March 19th, when the Duke of Fife will preside.

Structural Alterations.

Then there have also been some structural improvements during the year which necessitated the closing of the hospital for two months, from the middle of July to the middle of September. These comprise a large and airy kitchen, fitted with the newest and most efficient apparatus for cooking by gas and steam, and tiled with ivory-white tiles and paved with hard-pressed terra-cotta. A scullery, two larders, two store-rooms, a housekeeper's room, and a large servants' hall, all fitted with electric light, have been provided in the basement. The new workshop for the engineer, next to the boiler house, over which a fireproof floor has been laid, has been finished. The new sanitary annexe for the south wing has also been completed and is in use. On these necessary improvements the sum of £12,000 has been expended.

Some Statistics.

As to the number of patients treated, of course the closing of the wards makes a considerable difference, and the figures cannot be compared with those of last year, a difference of some thousands in each department being recorded, e.g., there were fewer new patients by nearly 6,000, and out-patient attendances were 8,000 less than in 1901. Neither would it be fair to compare the average cost per bed and per patient, the former being £6 and the latter nearly £2 higher than last year.

The annual subscriptions, which had slightly fallen off last year, again show a deficiency, being £2,319, whereas last year they were £2,339, and the year before £2,360. The total receipts, however, exceed those of last year by over £5,000. The difference is partly accounted for by the amount received in legacies, viz., over £8,000, and £2,025 has been paid from the David Hughes estates, divided among four hospitals, of which Charing Cross is one. The crew of H.M.S. *Illustrious* sent a donation of £30, through their chaplain, the Rev. J. H. de Vitre, in memory of their comrades who had died during the last commission. The amount received in response to the special appeal was £292 in excess of that received last year, being £3,298 12s. 11d.

New Developments.

Two new departments have been opened, an ophthalmic department for out-patients, to which Mr. W. Treacher

Collins, F.R.C.S., has been appointed Surgeon as well as Lecturer on Ophthalmology at the medical school; and secondly, a department for the treatment of lupus by the Finsen light. A complete apparatus has been installed, and the department is placed under Dr. Galloway, Physician for Skin Diseases. Special mention is made in the report of the excellent work done by the X-ray department, under the charge of Mr. Mackenzie Davidson.

The Convalescent Home has had several benefactions, including £100 from the Mansion House War Fund, towards maintaining the beds set apart for soldiers invalided from the South African War.

The report and accounts were adopted, and a long list of resolutions, chiefly of thanks to the various officers of the hospital, followed.

At twenty-two minutes past two the CHAIRMAN rose, congratulating the Court on the saving of three minutes in the duration of the meeting.

